# Supplementary material for: Deciphering the regulatory landscape of fetal and adult γδ T‐cell development at single‐cell resolution
Source: EMBO J. 2020 Jun 8;39(13):e104159. doi: 10.15252/embj.2019104159 (PMC7327493; doi:10.15252/embj.2019104159)
Supplement: Supplementary file 1 — Appendix [file EMBJ-39-e104159-s001.docx]

Appendix

Deciphering the Regulatory Landscape of Fetal and Adult γδ T cell Development at Single-cell Resolution

Sagar^1^, Maria Pokrovskii^2^, Josip S. Herman^1,3,4^, Shruti Naik^5^, Elisabeth Sock^6^, Patrice Zeis^1,3,4^, Ute Lausch^7^, Michael Wegner^6^, Yakup Tanriver^7,8^, Dan R. Littman^2,9^, Dominic Grün^1,10*^

Table of contents

Appendix Figure S1………………………………………………………………………………….2

Appendix Figure S2………………………………………………………………………………….3

Appendix Figure S3………………………………………………………………………………….4

Appendix Figure S4………………………………………………………………………………….6

Appendix Figure S5………………………………………………………………………………….7

Appendix Figure S6………………………………………………………………………………….8

**Appendix Figure S1. Integrating scRNA-seq datasets of γδ T cells from the blood, lymph nodes and thymus.**

A t-SNE representation based on transcriptome similarities showing 5 clusters identified by RaceID3 algorithm in the combined dataset from the blood and lymph nodes.

B Dot plot showing key marker genes differentially expressed in the clusters of the combined dataset from the blood and lymph nodes.

C UMAP representation showing the expression of key marker genes in the integrated dataset from the thymus, blood and lymph nodes.

**Appendix Figure S2. Gene network modules characterizing different stages of fetal and adult early thymopoiesis and γδ T cell differentiation.**

A,B Genes contained in fetal (A) and adult (B) gene network modules recovered using the GENIE3 algorithm.

**Appendix Figure S3. Characterization of γδ T cells in *Sox13* and *Maf* KO mice.**

A FACS plots showing CD24^+^ and CD24^-^ γδ T cells sorted from fetal and adult WT and *Sox13* KO thymi.

B FACS plots showing CD24^+^ and CD24^-^ γδ T cells sorted from fetal and adult *Maf^fl/fl^* and *Maf^fl/fl^;Il7ra^cre^* thymi.

C Heatmap showing selected differentially expressed genes between immature γδ T cells from the fetal WT and *Maf* KO thymi (adjusted *P* < 0.05). Differentially expressed genes were sorted by decreasing fold-change.

D Boxplots showing the expression of *Sox13* and *Rorc* in immature WT and *Maf* KO adult thymi. Each point represents a cell. Boxes indicate the first and the third quartiles. The vertical line indicates the median. Whiskers are extended to the extreme data points.

E Heatmap showing the differentially expressed genes between immature γδ T cells from the adult WT and KO thymi (adjusted *P* < 0.05, fold-change > 2). Differentially expressed genes were sorted be decreasing fold-change.

F GSEA of differentially expressed genes between immature γδ T cells from adult *Maf^fl/fl^* and *Maf^fl/fl^;Il7ra^cre^* thymi. The bar chart shows the normalized enrichment score (NES) and highlights the p-value.

G Fraction of RORγt^+^ γδ T cells in the skin-draining lymph nodes as well as in the skin of 6-weeks old *Maf* KO and WT mice.

H RT-PCR showing the IL-17a mRNA levels in the skin of the *Maf* KO and WT mice after 6 consecutive days of IMQ treatment (n=1 independent experiment, three adult mice each genotype). Bars represent the mean values and error bars indicate standard error of mean (SEM).

I Intracellular staining showing the quantification of RORγt^+^ γδ T cells in the skin of the *Maf* KO and WT mice after 6 days of consecutive IMQ application.

**Appendix Figure S4. *Rorc* is the endpoint of γδT17 effector differentiation program in the thymus.**

A Scheme showing the experimental design and scRNA-seq pipeline for the analysis of *Rorc* KO mice.

B FACS plots showing CD24^+^ and CD24^-^ γδ T cells sorted from fetal and adult WT and *Rorc* KO thymi.

C t-SNE representation showing the sorted cell types from WT and *Rorc* KO fetal thymi (n=1 independent experiment, three embryos from one female mouse per genotype). Grey color represents DN and CD25^+^ γδ T cells from the fetal WT data shown in **Fig 1C**. Note that cells expressing *Rorc*, *Il17a* and *Il17f* are absent in the KO mice (orange box).

D t-SNE representation highlighting the expression of *Sox13* and *Maf*. Note that *Rorc* KO immature fetal γδ T cells do not downregulate *Sox13* and *Maf* expression and intermingle with WT cells.

E Close-up of the t-SNE representation indicating that cells expressing *Sox13*, *Maf* and *Il17f* are absent form *Rorc* KO fetal thymi.

F t-SNE representation showing the sorted cell types from WT and *Rorc* KO adult thymi (n=2 independent experiments from two female mice per genotype). Grey color represents DN and CD25^+^ γδ T cells from the adult WT data shown in **Fig 1F**. Note that immature KO cells clustered separately from WT (black box) and that the mature *Rorc*^+^ γδ T cell compartment lacks KO cells (orange box).

G Close-up of the t-SNE representation showing the expression of *Sox13*, *Maf* and *Il17re* in immature γδ T cells. Note that the expression of *Sox13* remains unaffected while *Maf* expression is downregulated in the *Rorc* KO cells. *Il17re*^+^ cells are completely absent in the *Rorc* KO adult thymi.

H Close-up of the t-SNE representation showing the expression of *Sox13*, *Maf* and *Il17re* in mature γδ T cells. Note that mature γδT17 cells are completely absent in the *Rorc* KO adult thymi.

I Bar plot depicting the differentially expressed genes in immature γδ T cells between the *Rorc* KO and WT adult thymi. (purple: upregulated genes, green: downregulated genes, adjusted *P* < 0.05).

**Appendix Figure S5. Low-dimensional γδ T cell differentiation manifold remains stable across different perplexity values of dimensional reduction using t-SNE.**

A t-SNE representation based on transcriptome similarities of fetal γδ data showing 30 clusters at different perplexity values. Note that the structure of the data remained fairly stable across different values, and the default value (set to 30) of the RaceID3 algorithm was used to represent the dataset.

B t-SNE representation based on transcriptome similarities of adult γδ data showing 24 clusters at different perplexity values. Similar to the fetal data, that the structure of the adult dataset remained stable across different values, and the default value was used to represent the dataset. A similar analysis was performed for all the other datasets and the default value (set to 30) was used to represent all the other datasets in the manuscript where dimensionality reduction was performed using t-SNE.

**Appendix Figure S6. Freezing does not affect the transcriptome and the subsets of γδ thymocytes.**

A t-SNE representation based on transcriptome similarities of fetal γδ thymocytes from fresh WT thymocytes (green), frozen γδ T cells from *Maf* (red) and *Sox13* (blue) WT littermate controls. Note that there is no substantial effect of freezing on the transcriptome of γδ T cells. Fresh WT γδ T cells intermingle well with frozen *Maf* and *Sox13* WT γδ T cells and all major γδ subsets from the frozen material were recovered.

B t-SNE representation based on transcriptome similarities of adult γδ thymocytes from fresh WT thymocytes (green), frozen γδ T cells from *Maf* (red) and *Sox13* (blue) WT littermate controls. As in the fetus, fresh adult WT γδ T cells intermingle well with frozen *Maf* and *Sox13* WT γδ T cells and all major γδ subsets from the adult frozen thymi were recovered.
