# Supplementary material for: Deciphering the regulatory landscape of fetal and adult γδ T‐cell development at single‐cell resolution
Source: EMBO J. 2020 Jun 8;39(13):e104159. doi: 10.15252/embj.2019104159 (PMC7327493; doi:10.15252/embj.2019104159)
Supplement: Supplementary file 2 — Expanded View Figures PDF [file EMBJ-39-e104159-s002.pdf]

Expanded View Figures

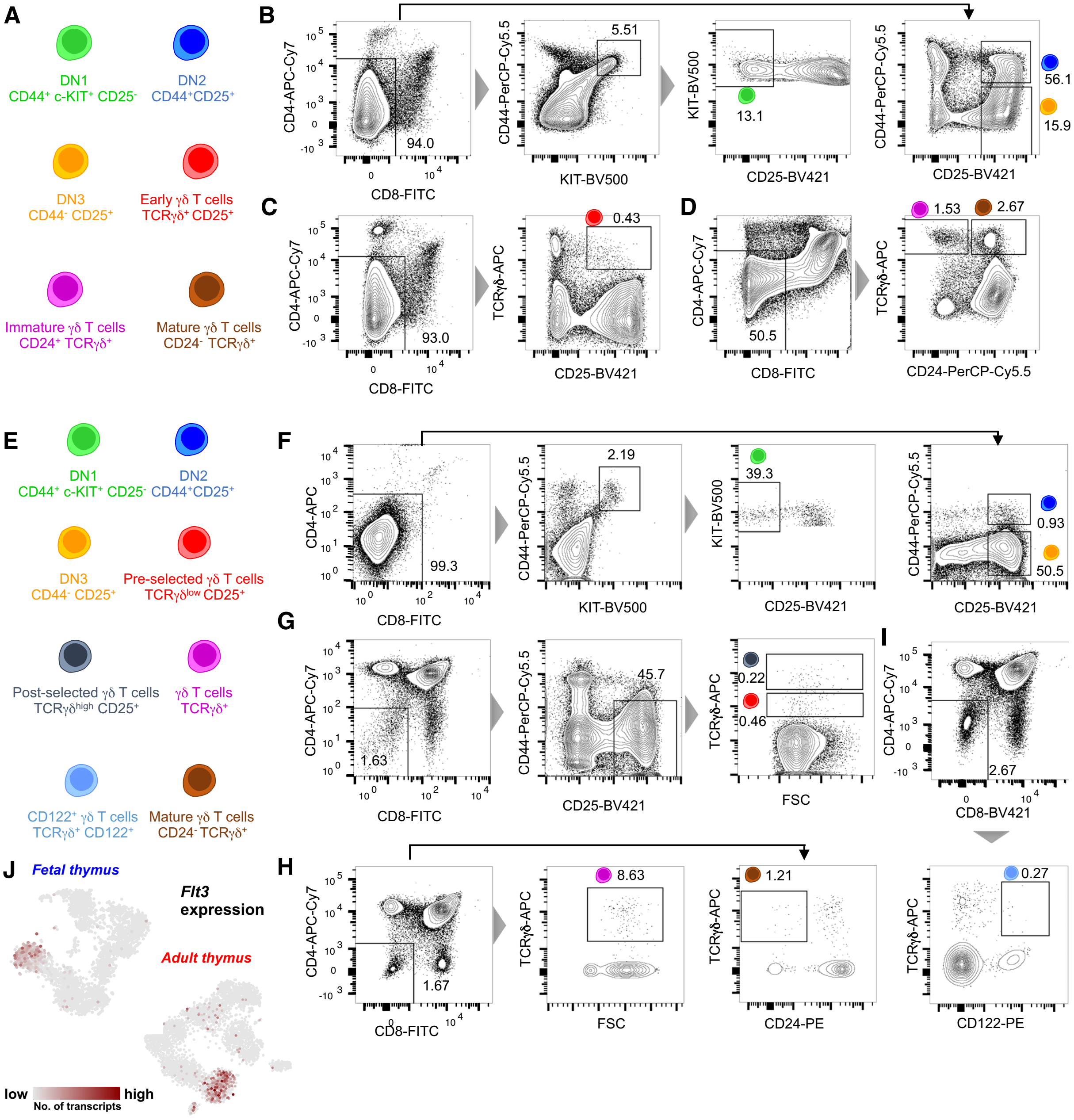

Figure EV1. FACS strategy for scRNA-seq experiments.

A Sketch showing different cell types sorted for scRNA-seq experiments and the associated cell surface markers used from the fetal thymus.

B–D FACS plots showing the gates used for sorting (B) c-KIT$^+$ DN1, DN2, and DN3 T cells, (C) CD25$^+$ γδ T cells, and (D) CD24$^+$ immature and CD24$^-$ mature γδ T cells from fetal thymus.

E Sketch showing different cell types sorted for scRNA-seq experiments and the associated cell surface markers used from the adult thymus.

F FACS plots showing the gates used for sorting c-KIT$^+$ DN1, DN2, and DN3 T cells from the adult thymus. Note that before sorting DN1-DN3 populations, thymocytes were enriched for DN populations using magnetic cell enrichment.

G, H FACS plots showing the gates used for sorting (G) pre-selected and post-selected γδ T cells and (H) pan γδ T cells and CD24$^-$ mature γδ T cells from the adult thymus. Note that > 98% of the pan γδ T cells are immature γδ T cells.

I FACS plots showing the gates used for sorting CD122$^+$ γδ T cells from the adult thymus

J t-SNE representation of the fetal and adult data showing the expression of *Flt3*.

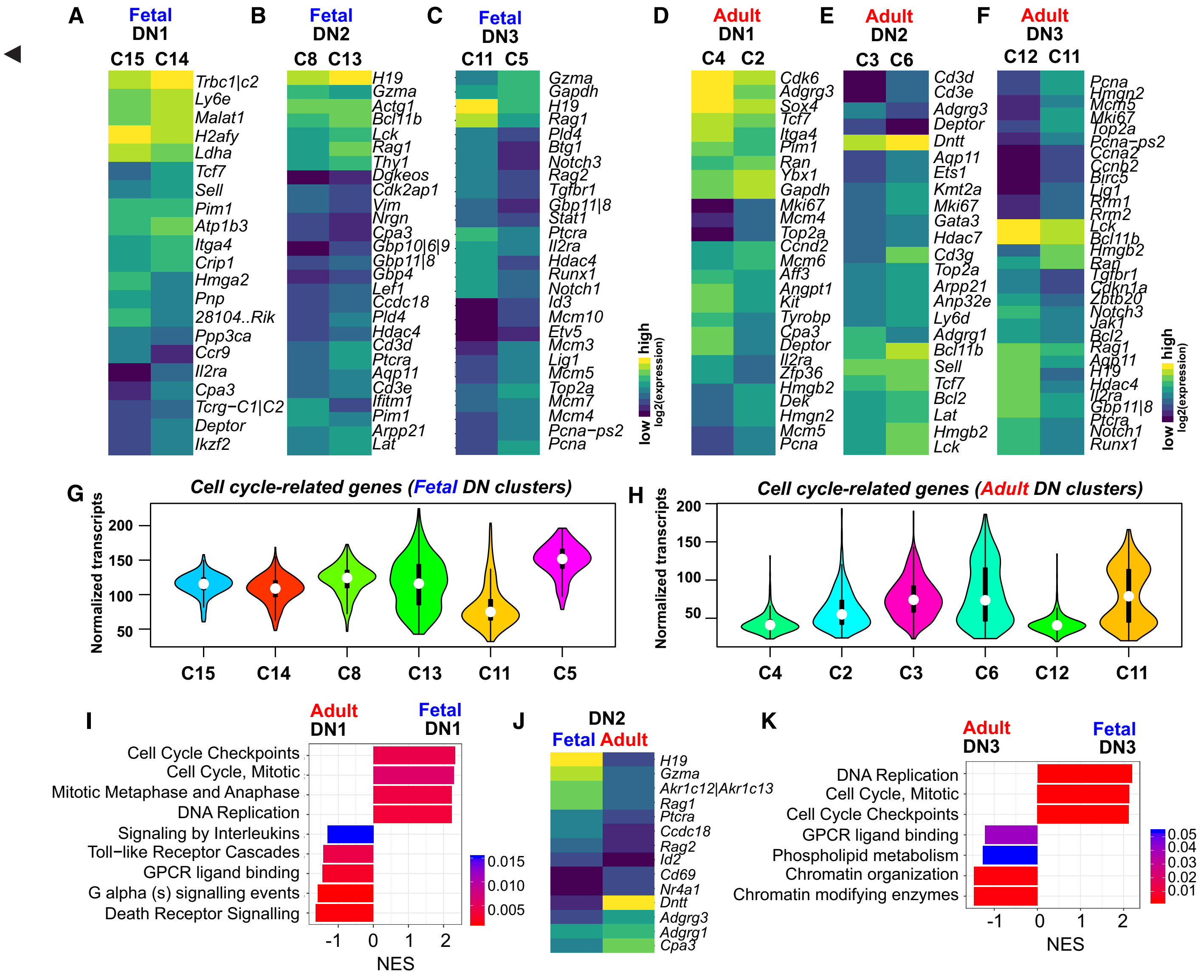

Figure EV2. Transcriptional heterogeneity in the double negative T-cell progenitors from the fetal and adult thymus.

A–F Heatmap showing the differentially expressed genes between (A) fetal c-KIT^{+} DN1 clusters, (B) fetal DN2 clusters, (C) fetal DN3 clusters, (D) adult c-KIT^{+} DN1 clusters, (E) adult DN2 clusters, and (F) adult DN3 clusters. Shortlisted genes had adjusted $P < 0.05$.

G, H Violin plots showing the aggregated normalized transcript counts for cell cycle-related genes in (G) fetal and (H) adult DN1–DN3 enriched clusters.

I GSEA of differentially expressed genes between fetal and adult c-KIT^{+} DN1s. The bar chart shows the normalized enrichment score (NES) and highlights the *P*-value.

J Heatmap showing the differentially expressed genes between fetal and adult DN2s (shortlisted genes had adjusted $P < 0.05$).

K GSEA of differentially expressed genes between fetal and adult DN3s. The bar chart shows the normalized enrichment score (NES) and highlights the *P*-value.

© 2020 The Authors

Figure EV3. Transcriptional heterogeneity of fetal and adult γδ T cells at various stages of differentiation.

A t-SNE representations of the fetal thymus dataset showing the weights for adult cluster 8 expressing high TCR signaling-related genes and cluster 9 expressing γδT17-biased genes. Weights were calculated using quadratic programming. Color scale represents weights on the scale of 0–1.

B, C t-SNE representation showing the expression of selected genes in (B) fetal and (C) adult γδ T-cell progenitors comprising immature and mature γδ T-cell compartments.

D t-SNE representation showing the expression of *Rorc*. Note that mature *Rorc*$^+$ cells exhibit mutually exclusive expression of *Scart1* and *Scart2*. *Scart1*$^+$ cells are *Zbtb16*$^+$.

E t-SNE representation of the adult thymus dataset showing the weights for fetal *Gzma* (cluster 1), *Rorc* (clusters 3 and 23) and *Il2rb* (cluster 6) clusters calculated using quadratic programming. Color scale represents weights on the scale of 0–1.

F UMAP representation showing the expression of key marker genes in the integrated fetal and adult dataset.

© 2020 The Authors

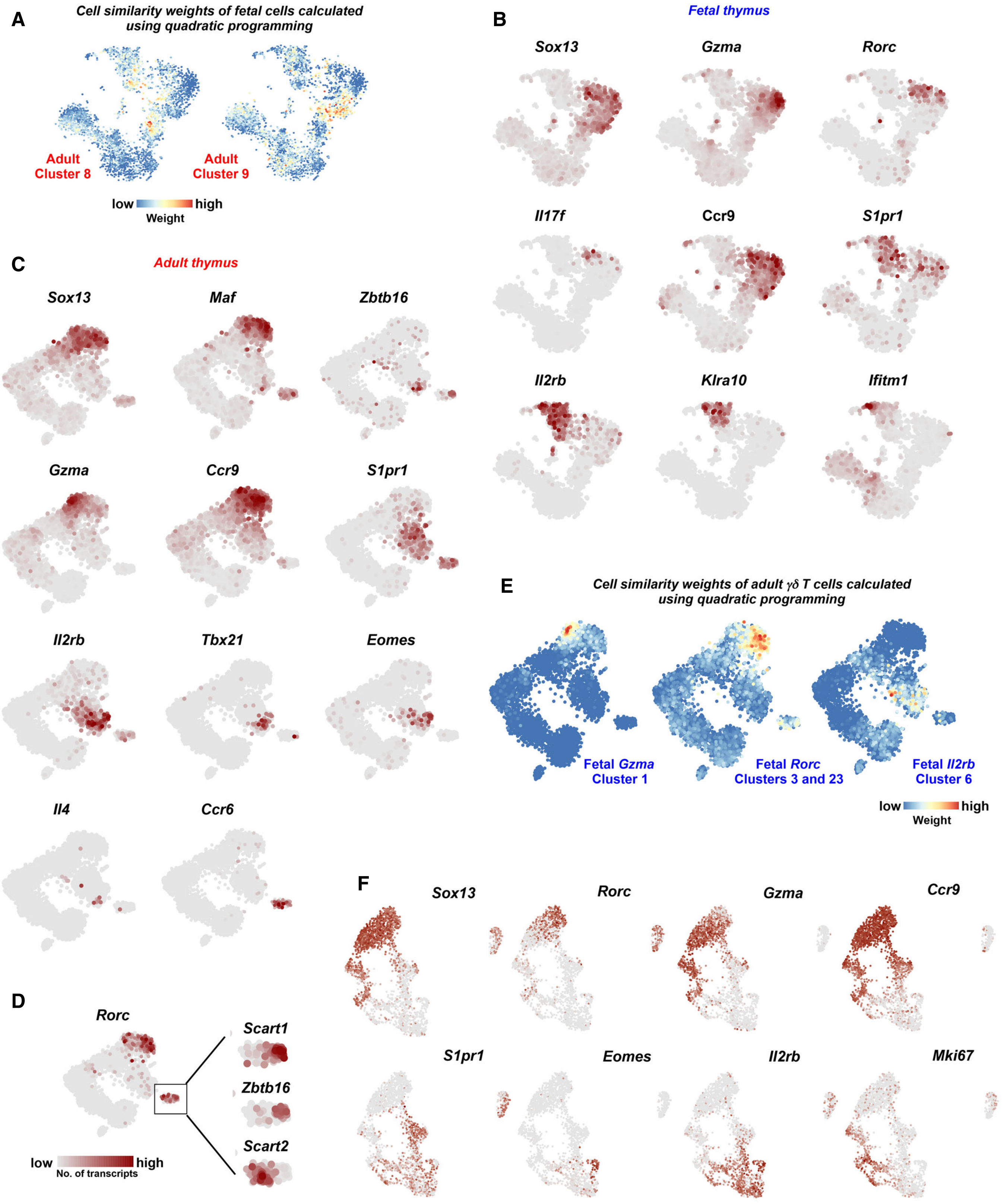

Figure EV3.

 © 2020 The Authors

Figure EV4. scRNA-seq of fetal and adult γδ T cells expressing different variable chains.

A, B t-SNE representation based on transcriptome similarities showing (A) fetal (*n* = 1 independent experiment, eight embryos from one female mouse) and (B) adult (*n* = 1 independent experiment from one 6-week-old female mouse) immature and mature γδ T-cell types sorted for different variable chains depicted in different colors. Cells from the original datasets are highlighted in gray.

C–H Pie charts showing the contribution of immature and mature (C) fetal $V\gamma1^+$, (D) fetal $V\gamma5^+$, (E) fetal pan $V\gamma4^+$, (F) adult $V\gamma1^+$, (G) adult pan $V\gamma4^+$, and (H) $V\gamma1^+V\delta6.3^+$ γδ T cells to the different sub-types in the immature ($CD24^+$) and mature ($CD24^-$) compartments in the thymus.

I, J t-SNE representation showing the expression of key marker genes in (I) fetal and (J) adult data.

© 2020 The Authors

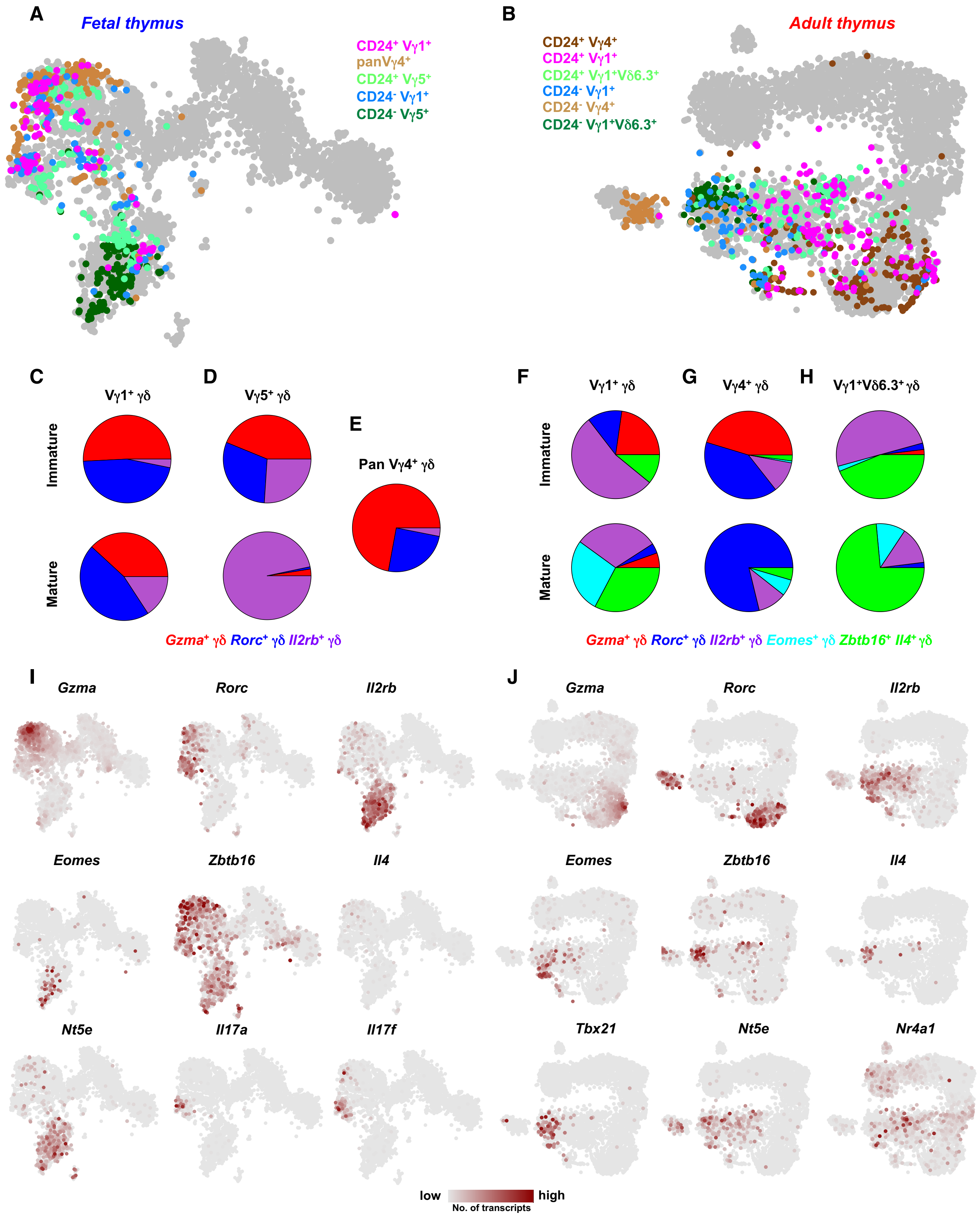

Figure EV4.

© 2020 The Authors

Figure EV5. Reconstruction of fetal and adult γδ T-cell differentiation trajectories identified co-regulated gene modules expressed at different stages of development.

A SOM of *z*-score-transformed, pseudo-temporal expression profiles along the fetal IL-17-producing γδ T-cell (γδT17) differentiation trajectory. The color-coding at the bottom indicates the cluster of origin. The SOM identified 17 different modules of co-regulated genes.

B Pseudo-temporal expression profiles of transcription factors, as well as receptors, cell surface markers, and secreted proteins with known and unknown functions activated during fetal γδT17 differentiation. *Y*-axis represents aggregated normalized counts of genes. *X*-axis represents the pseudo-temporal order. The lines indicate the pseudo-temporal expression values derived by a local regression of expression values across the pseudo-temporal order.

C SOM of *z*-score-transformed, pseudo-temporal expression profiles along the fetal IFN-γ-producing γδ T-cell differentiation trajectory. The SOM identified 20 different modules of co-regulated genes. The color-coding at the bottom indicates the cluster of origin.

D Pseudo-temporal expression profiles of transcription factors, as well as receptors, cell surface markers, and secreted proteins with known and unknown functions activated during fetal IFN-γ-producing γδ T-cell differentiation. *Y*-axis represents aggregated normalized counts of genes. *X*-axis represents the pseudo-temporal order. The lines indicate the pseudo-temporal expression values derived by a local regression of expression values across the pseudo-temporal order.

E List of transcription factors, receptors, cell surface markers, and secreted proteins upregulated during fetal γδT17 differentiation along the pseudo-temporal order shown in Fig EV5A.

F List of transcription factors, receptors, cell surface markers, and secreted proteins upregulated during fetal IFN-γ-producing γδ T-cell differentiation along the pseudo-temporal order shown in Fig EV5C.

G, H SOM of *z*-score-transformed, pseudo-temporal expression profiles along the adult (G) γδT17 and (H) IFN-γ-producing γδ T-cell differentiation trajectories. The color-coding at the bottom indicates the cluster of origin. The SOM identified 22 and 31 different modules of co-regulated genes, respectively.

I, J Pseudo-temporal expression profiles of transcription factors, as well as receptors, cell surface markers, and secreted proteins with known and unknown functions activated during (I) adult γδT17 and (J) IFN-γ-producing γδ T-cell differentiation. *Y*-axis represents aggregated normalized counts of genes. *X*-axis represents the pseudo-temporal order. The lines indicate the pseudo-temporal expression values derived by a local regression of expression values across the pseudo-temporal order.

K, L GSEA of differentially expressed genes between (K) fetal and (L) adult γδT17 and IFN-γ-producing γδ T cells. The bar chart shows the normalized enrichment score (NES) and highlights the *P*-value.

M t-SNE representations of fetal an adult data showing the aggregated expression of common histone-modifying factors identified in fetal and adult γδT17 modules. Note the higher expression of the listed factors in DN3 cells undergoing recombination and *Rorc*$^{+}$ γδT17 lineage cells.

© 2020 The Authors

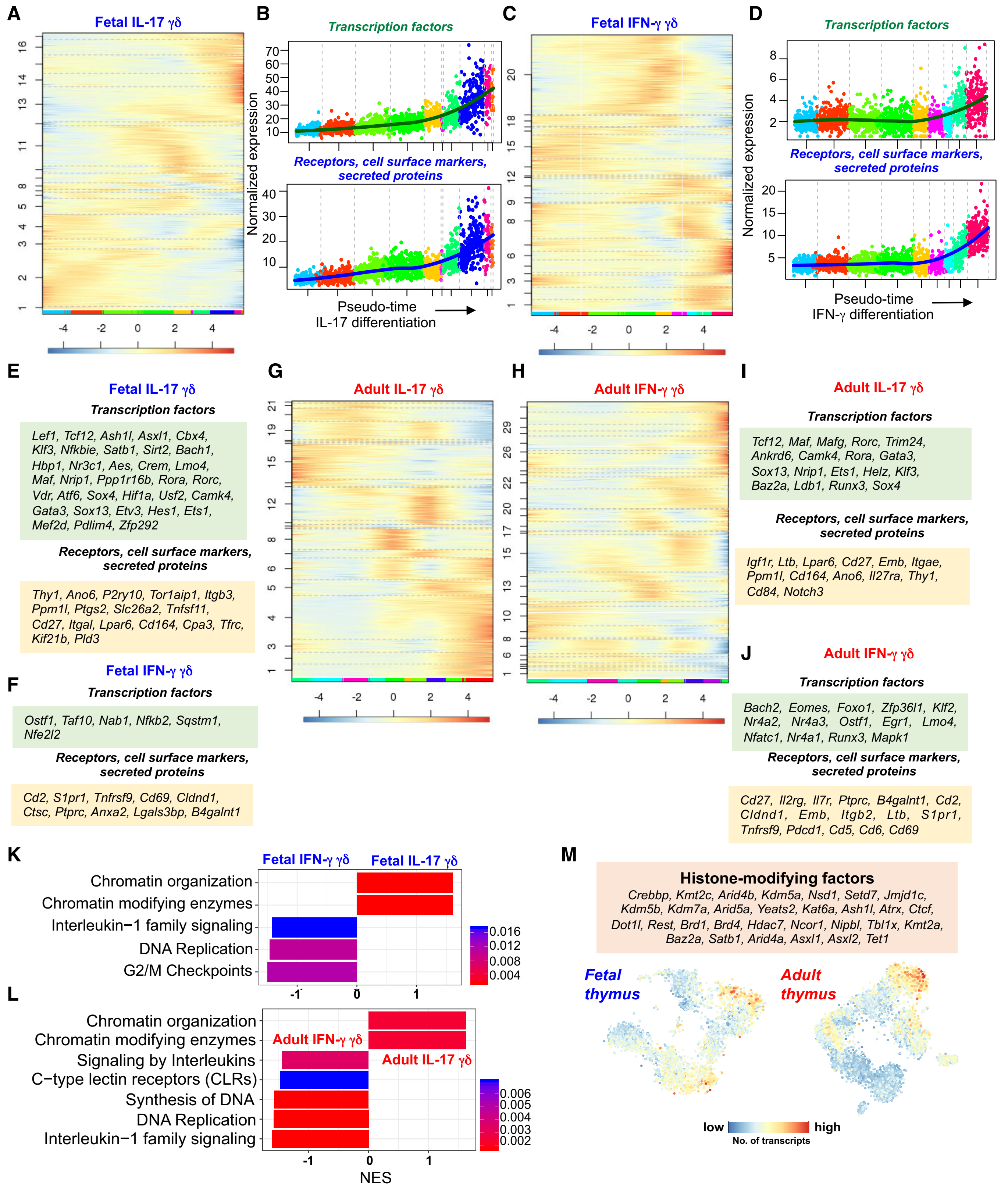

Figure EV5.

 © 2020 The Authors
